# High rates of viral suppression in adults and children with high CD4+ counts using a streamlined ART delivery model in the SEARCH trial in rural Uganda and Kenya

**DOI:** 10.7448/IAS.20.5.21673

**Published:** 2017-07-21

**Authors:** Dalsone Kwarisiima, Moses R. Kamya, Asiphas Owaraganise, Florence Mwangwa, Dathan M. Byonanebye, James Ayieko, Albert Plenty, Doug Black, Tamara D. Clark, Bridget Nzarubara, Katherine Snyman, Lillian Brown, Elizabeth Bukusi, Craig R. Cohen, Elvin H. Geng, Edwin D. Charlebois, Theodore D. Ruel, Maya L. Petersen, Diane Havlir, Vivek Jain

**Affiliations:** ^a^ Infectious Diseases Research Collaboration, Kampala, Uganda; ^b^ School of Medicine, Makerere University College of Health Sciences, Kampala, Uganda; ^c^ Kenya Medical Research Institute, Nairobi, Kenya; ^d^ Division of HIV, Infectious Diseases & Global Medicine, University of California, San Francisco, CA, USA; ^e^ Department of Obstetrics, Gynecology & Reproductive Sciences, University of California, San Francisco, CA, USA; ^f^ Division of Infectious Diseases, Department of Pediatrics, University of California, San Francisco, CA, USA; ^g^ Department of Biostatistics and Epidemiology, Berkeley School of Public Health, Berkeley, CA, USA

## Abstract

**Introduction**: The 2015 WHO recommendation of antiretroviral therapy (ART) for all HIV-positive persons calls for treatment initiation in millions of persons newly eligible with high CD4+ counts. Efficient and effective care models are urgently needed for this population. We evaluated clinical outcomes of asymptomatic HIV-positive adults and children starting ART with high CD4+ counts using a novel streamlined care model in rural Uganda and Kenya.

**Methods**: In the 16 intervention communities of the HIV test-and-treat Sustainable East Africa Research for Community Health Study (NCT01864603), all HIV-positive individuals irrespective of CD4 were offered ART (efavirenz [EFV]/tenofovir disoproxil fumarate + emtricitabine (FTC) or lamivudine (3TC). We studied adults (≥fifteen years) with CD4 ≥ 350/μL and children (two to fourteen years) with CD4 > 500/μL otherwise ineligible for ART by country guidelines. Clinics implemented a patient-centred streamlined care model designed to reduce patient-level barriers and maximize health system efficiency. It included (1) nurse-conducted visits with physician referral of complex cases, (2) multi-disease chronic care (including for hypertension/diabetes), (3) patient-centred, friendly staff, (4) viral load (VL) testing and counselling, (5) three-month return visits and ART refills, (6) appointment reminders, (7) tiered tracking for missed appointments, (8) flexible clinic hours (outside routine schedule) and (9) telephone access to clinicians. Primary outcomes were 48-week retention in care, viral suppression (% with measured week 48 VL ≤ 500 copies/mL) and adverse events.

**Results**: Overall, 972 HIV-positive adults with CD4+ ≥ 350/μL initiated ART with streamlined care. Patients were 66% female and had median age thirty-four years (IQR, 28–42), CD4+ 608/μL (IQR, 487–788/μL) and VL 6775 copies/mL (IQR, <500–37,003 c/mL). At week 48, retention was 92% (897/972; 2 died/40 moved/8 withdrew/4 transferred care/21/964 [2%] were lost to follow-up). Viral suppression occurred in 778/838 (93%) and 800/972 (82%) in intention-to-treat analysis. Grade III/IV clinical/laboratory adverse events were rare: 95 occurred in 74/972 patients (7.6%). Only 8/972 adults (0.8%) switched ART from EFV to lopinavir (LPV) (*n* = 2 for dizziness, *n *= 2 for gynaecomastia, *n *= 4 for other reasons). Among 83 children, week 48 retention was 89% (74/83), viral suppression was 92% (65/71) and grade III/IV adverse events occurred in 4/83 (4.8%).

**Conclusions**: Using a streamlined care model, viral suppression, retention and ART safety were high among asymptomatic East African adults and children with high CD4+ counts initiating treatment.

**Clinical Trial Number**: NCT01864603

## Introduction

Global guidelines now endorse antiretroviral therapy (ART) for all HIV-infected individuals [1] to improve broad clinical outcomes [2,3], reduce transmission [4] and aggressively impact the course of the HIV epidemic [5]. An estimated 16 million persons are now on ART worldwide [6], but much greater ART access, centred in sub-Saharan Africa, needs to be achieved to reach global goals [7]. This urgent need to expand ART delivery occurs at a time of global resource constraints [8], making it imperative that we design, test and implement highly efficient and effective models of care for ART delivery.

As ART access and availability expand, large numbers of patients previously ineligible for therapy – particularly patients with high CD4+ T-cell counts and asymptomatic disease – are entering care. Older models of care designed during the AIDS emergency response over a decade ago are not optimally efficient for either patients or the health system. Until recently, most care delivery systems have offered “one-size-fits-all” approaches with similar services for both sicker and healthier patients. These models do not capitalize on the fact that healthier patients with high CD4+ counts above 350 cells/μL can be served with streamlined care systems that may be more effective for the patient and highly efficient for the health system [9].

A large and growing body of literature has evaluated many single-component interventions designed to improve clinic efficiency and offer “differentiated care” interventions including pharmacy refill programmes [10,11], nurse-driven triage systems [12–14], mobile phone outreach [15–17] and community navigation services [18,19]. However, literature on combination interventions – packages of interventions that together comprise a system of care delivery – is limited.

Our group previously tested a streamlined ART delivery system in a single Ugandan clinic and demonstrated strong retention in care and viral suppression among patients with CD4+ counts above Ugandan guideline threshold (CD4 > 350) [20]. This model was adapted as part of the intervention in the SEARCH Study (Sustainable East Africa Research for Community Health: NCT01864603), a large-scale HIV test-and-treat study assessing whether a strategy of universal HIV testing and ART delivery lowers HIV incidence and improves health outcomes. In this report, we evaluate one-year outcomes of retention, viral suppression and safety of our streamlined care model across a series of rural communities in Uganda and Kenya.

## Methods

### Ethics statement

This study was approved by ethical review boards of Makerere University, Uganda National Council of Science and Technology (Kampala, Uganda), Kenya Medical Research Institute (Nairobi, Kenya) and the University of California, San Francisco (USA). Participants were consented for study participation.

### Study setting

This is a 48-week analysis of clinical and virologic outcomes of Ugandan and Kenyan HIV-infected adults (age ≥ fifteen years) and children (age two to fourteen years) with high CD4+ cell counts, not qualifying for ART by current country guidelines, who received ART using a patient-centred streamlined model of care.

The SEARCH Study is an ongoing community cluster randomized trial of a universal HIV “test-and-treat” strategy in 32 rural Ugandan and Kenyan communities (NCT01864683). In 16 SEARCH intervention communities, all community members (adults and children) were offered annual HIV testing via community health fairs. Health fair non-attendees received home visits during which HIV testing was offered [21]. Point-of-care CD4+ cell counts were determined in all HIV-positive persons (PIMA, Alere). Participants, upon HIV diagnosis at a community health fair or during in-home testing, were immediately given appointments at the local clinic for immediate evaluation in <1 week for ART initiation. Pregnant women and persons with CD4 < 200/μL received appointment dates within 2 days. Adult patients or guardians of paediatric patients were given the clinic’s mobile phone number and encouraged to call with questions about appointments, symptoms or any other reason. This phone was staffed 24 h/day. Patients were also offered a single small transport reimbursement upon presentation to the first clinic visit.

### ART initiation and monitoring

In clinics, ART eligibility was first assessed using Uganda- and Kenya-specific guidelines between June 2013 and June 2014. In both countries, at the start of study, persons were eligible for ART if a WHO Stage 3 or 4 condition was diagnosed, if CD4+ count was <350/μL, or if CD4 > 350 and patient had (1) tuberculosis, (2) hepatitis B virus infection or (3) pregnancy. Individuals meeting country ART initiation guidelines received medications through the existing government-sponsored programme. Patients ineligible for ART by country guidelines were offered participation under informed consent described here. For children, informed consent was obtained from a legal guardian.

ART was provided by the study. For adults, this included efavirenz (EFV) plus tenofovir disoproxil fumarate (TDF) co-formulated either with emtricitabine (i.e. FTC/TDF, Truvada) or with lamivudine (i.e. FTC/3TC), consistent with national guidelines. For children, ART included abacavir/3TC with either EFV or nevirapine, or alternately, TDF with either FTC or 3TC, consistent with national guidelines. Following ART initiation, adult and child participants had clinic visits at weeks 0, 4, 12, 24, 36 and 48. Safety laboratory monitoring (creatinine, aspartate and alanine transferase and haemoglobin) was performed at 24 and 48 weeks and results graded by the DAIDS laboratory adverse event scale [22]. Clinicians asked patients about adverse symptoms or clinical events since their last visit; they also recorded events volunteered by participants at regularly scheduled or off-schedule visits.

### Patient-centred streamlined care model for clinics

Part of the SEARCH Study HIV test-and-treat intervention strategy is a multicomponent streamlined ART delivery model (Table 1). This streamlined care model is designed around three central principles: (1) reducing structural barriers to care, (2) improving relationships between patients and the clinic and (3) enhancing patient and clinician knowledge of HIV and ART.

**Table 1.** Features of streamlined care designed to improve visit attendance, ART adherence and clinic efficiency.

	Reduced structural barriers to care			Improved relationship with clinic		Enhanced attitudes and knowledge		
	Waiting time	Cost	Convenience	Direct access to clinicians	Respectful interactions	Improve motivation	Improve HIV and ART knowledge	Reduce stigma
**HIV care delivery and retention**								
Nurse triage and care	**•**							
Three month visit for stable patients	**•**	**•**	**•**			**•**		
Multi-disease chronic care model		**•**	**•**				**•**	**•**
Friendly clinic staff					**•**	**•**		**•**
Appointment reminders				**•**		**•**		
Tiered tracking for clinic no-show			**•**	**•**				
Viral load counselling							**•**	
Flexible clinic hours	**•**		**•**	**•**		**•**		**•**
Phone access to clinic provider^A^	**•**	**•**	**•**	**•**	**•**	**•**	**•**	**•**

^A^Provided for questions related to symptoms/clinical problems, ART, scheduling or logistics of appointments or other concerns. • Indicates that the streamlined care intervention targets the area for clinical operations improvement.

Upon linkage to the clinic, adult and paediatric participants received care in the streamlined care model. This model was designed to reduce clinic inefficiencies that arise when staffing and services needed for HIV-positive patients with symptomatic, complex HIV disease are applied uniformly to patients with early stage, asymptomatic HIV disease. In our clinics, HIV care was part of a chronic disease care model offering joint evaluation and management of hypertension, diabetes and general medical conditions. All patients receiving HIV care, hypertension/diabetes and medical care were served with identical processes.

Structural barriers to care were targeted in several ways. First, our streamlined care model used a nurse-driven triage and care system (Table 1) that aimed to reduce wait times by fostering rapid, focused clinic visits. Nurses evaluated patients for ART side effects, assessed ART adherence and, if needed, consulted with a physician for advice. Periodically, nurses also dispensed ART and performed phlebotomy while at other times, patients received ART at the clinic pharmacy and saw a phlebotomist. Wait times were decreased by giving patients three-month ART refills rather than standard one- or two-month refills. Second, we aimed to reduce opportunity costs (e.g. missing work and transportation costs) to patients for attending clinic visits in two ways. Our clinical model offered multi-disease services including HIV, hypertension and diabetes care. We hypothesized that this could allow patients with multiple diagnoses to receive services for multiple conditions efficiently, rather than in separate visits, reducing transportation and opportunity costs of time away from work. Additionally, longer three-month ART refills were designed to reduce opportunity costs of making clinic visits. Third, our streamlined care model aimed to improve convenience for patients. Patients were offered the clinic’s mobile phone number and encouraged to call for appointment rescheduling or to clarify appointment dates in order to avoid missed visits. Appointment reminder phone calls were made to all patients 1 week prior to appointments. Further, patients expressing difficulty attending clinic visits during normal operating hours were accommodated with off-hours visits as needed. Finally, patients who missed visits received a tiered series of re-engagement interventions, including a phone contact, home visit and facilitated transport to return to clinic.

Improved relationships between patients and clinics were prioritized in several ways (Table 1). Clinic staff received training in methods of fostering a friendly and patient-centred atmosphere in clinic, and during all communications via the phone hotline. Training consisted of role-play scenarios and didactic lectures and was augmented with monthly team meetings to discuss challenging situations and share “success stories” recorded by staff in diaries. Further, the care model also featured appointment reminder phone calls and access to a clinic phone hotline for questions, both of which we hypothesized would strengthen patients’ feeling of connection and caring from the clinic.

Finally, our streamlined care model aimed to improve both clinicians’ and patients’ knowledge of ART and HIV disease so that patient motivation to attend visits would be higher, and stigma experienced by patients in coming to clinic would be reduced (Table 1). We performed viral load (VL) testing and provided interpretation of results and structured counselling to all patients. Clinic staff received training on VL counselling methods, using scripts containing scenarios of both viral suppression and non-suppression to enhance their knowledge of HIV disease and the effects of ART. Clinician training emphasized methods to transmit this knowledge directly to patients to increase their understanding of both HIV and ART, and to promote motivation and ART adherence. Telephone access, noted above, was also hypothesized as a vehicle for clinicians to educate patients on HIV and ART topics. Lastly, the streamlined care model was designed to reduce stigma in two central ways. First, by co-locating HIV services with hypertension, diabetes and other services, we sought to mitigate stigma that could be attached to attending a clinic known primarily for HIV services (i.e. “HIV clinic”). Second, as noted, the patient-centred and friendly clinic atmosphere was designed to avoid negative, judgmental or adverse clinician–patient interactions that are a known cause of stigma. Supplementary Appendix A summarizes features of the SEARCH streamlined care model, with contrast to features of the care model in use in SEARCH Study clinics prior to initiation of the study. Paediatric-specific adaptations to the streamlined care model are also described (Supplementary Appendix A).

### Study outcomes

We report clinical and laboratory outcomes among these cohorts of adults receiving ART through streamlined care. Retention in care was measured as the per cent of participants attending clinic visits at 12, 24 and 48 weeks, and loss to follow-up was defined as a patient who missed a visit and subsequently did not reappear. The virologic outcome of the study was HIV RNA suppression, defined as the per cent of participants with VL < 500 copies/mL at week 48. ART safety outcomes were assessed as the per cent of participants with grade III/IV clinical or laboratory adverse events (DAIDS scale) [22]. To assess utilization of the non-communicable disease services offered in our streamlined care model in the Uganda clinics, we determined the proportion of adult patients who had a visit for either diabetes or hypertension care. On enrolment in the study, patients were given a phone number to contact a clinician and told they could call 24 h per day for any problem, question or concern. A staff clinician from each clinic held this phone, responded to calls promptly and recorded, for each call, the identity of the caller, the time of the call, the total duration of the call, the reason(s) for the call and their response actions to the call. To assess utilization of the clinician access telephone hotline, we tabulated in the Uganda clinics the numbers of phone calls received, and for each call, the time of day, duration, reason for call and what actions clinicians took in response to the call.

## Results

### Baseline characteristics of adults and children

Overall, 972 adults with CD4 ≥ 350/μL and who were ineligible for government-provided therapy initiated ART (*n *= 411 [West Uganda], *n *= 99 [East Uganda] and *n *= 462 [Kenya]; Table 2). Median age was thirty-four years (IQR, 28–42), 66% of participants were female, 56% worked in farming/agriculture and 13% had higher than primary education. Median baseline CD4+ count was 608/μL (IQR, 487–788/μL), and median baseline VL was 6775 copies/mL (IQR, <500–37,003; Table 2).

**Table 2.** Baseline characteristics of adult (*n* = 972) and child (*n* = 83) participants initiating ART at high CD4+ counts ≥350/μL

	Adults (≥fifteen years)						Children (two–fourteen years)	
	**CD4+ 350–500/μL** (*n* = 269)		**CD4 > 500/μL** (*n* = 703)		**All adults** (*n* = 972)		**CD4 > 500/μL** (*n* = 83)	
Characteristic	*N*	%	*N*	%	*N*	%	*N*	%
**Region**								
West Uganda	104	38.7	307	43.7	411	42.3	26	31.3
East Uganda	21	7.8	78	11.1	99	10.2	11	13.3
Kenya	144	53.5	318	45.2	462	47.5	46	55.4
**Gender**								
Male	109	40.5	220	31.3	329	33.8	36	43.4
Female	160	59.5	483	68.7	643	66.2	47	56.6
**Age in years**								
<5	–	–	–	–	–	–	12	14.5
5–9	–	–	–	–	–	–	42	50.6
10–14	–	–	–	–	–	–	29	34.9
15–20	15	5.6	36	5.1	51	5.2	–	–
21–50	221	82.2	589	83.8	810	83.3	–	–
>50	33	12.3	78	11.1	111	11.4	–	–
**Educational status achieved**^A^								
No school	21	8.0	69	10.1	90	9.6	2	3.1
Primary	201	77.0	526	77.4	727	77.3	61	95.3
Secondary	29	11.1	65	9.6	94	10.0	1	1.6
Tertiary/Vocational	7	2.7	16	2.4	23	2.4	–	–
University	3	1.1	4	0.6	7	0.7	–	–
Post graduate	0	0.0	0	0.0	0	0.0	–	–
**Occupation**^B^								
Farmer	137	52.5	388	57.1	525	55.8	–	–
Fishing/Fishmonger	28	10.7	49	7.2	77	8.2	–	–
Shopkeeper/Market vendor	30	11.5	71	10.4	101	10.7	–	–
Household worker	6	2.3	42	6.2	48	5.1	–	–
Manual labour	14	5.4	21	3.1	35	3.7	–	–
Teacher	8	3.1	17	2.5	25	2.7	–	–
Student	6	2.3	18	2.6	24	2.6	–	–
Hotel/Restaurant worker	4	1.5	15	2.2	19	2.0	–	–
Transport	3	1.1	12	1.8	15	1.6	–	–
Other^C^	25	9.3	47	6.7	72	7.4	–	–
**Median baseline CD4+ count** [cells/μL] (IQR)	431 (396–467)		703 (587–864)		608 (487–788)		863 (662–1180)	
**Median baseline HIV RNA level** [copies/mL], (IQR)	21,124 (1530–82,006)		5075 (<500–23,620)		6775 (<500–37,003)		23,343 (8644–98,613)	
<10,000 c/mL	103	38.3	393	55.9	496	51.0	19	22.9
10,001–100,000 c/mL	98	36.4	194	27.6	292	30.0	31	37.3
≥100,000 c/mL	52	19.3	50	7.1	102	10.5	16	19.3
Not Available	16	5.9	66	9.4	82	8.4	17	20.5

^A^Educational status data available for 941/972 adults and 64/83 children. ^B^Occupation available for 941/972 adults. ^C^Includes healthcare, government, military or clerical worker, bar owner/worker, disabled, other job and no job.

A total of 83 children with CD4 ≥ 500/μL who were ineligible for government-provided therapy initiated ART (*n *= 26 [West Uganda], *n *= 11 [East Uganda] and *n *= 46 [Kenya]; Table 2). Median age was eight years (IQR, 6–11), 57% were female and 29/83 (35%) were orphaned. Median baseline CD4+ count was 863/μL (IQR, 662–1180/μL), and median baseline VL was 23,343 c/mL (IQR, 8644–98,613; Table 2).

### Adult retention in care and virologic suppression

**Figure 1.**
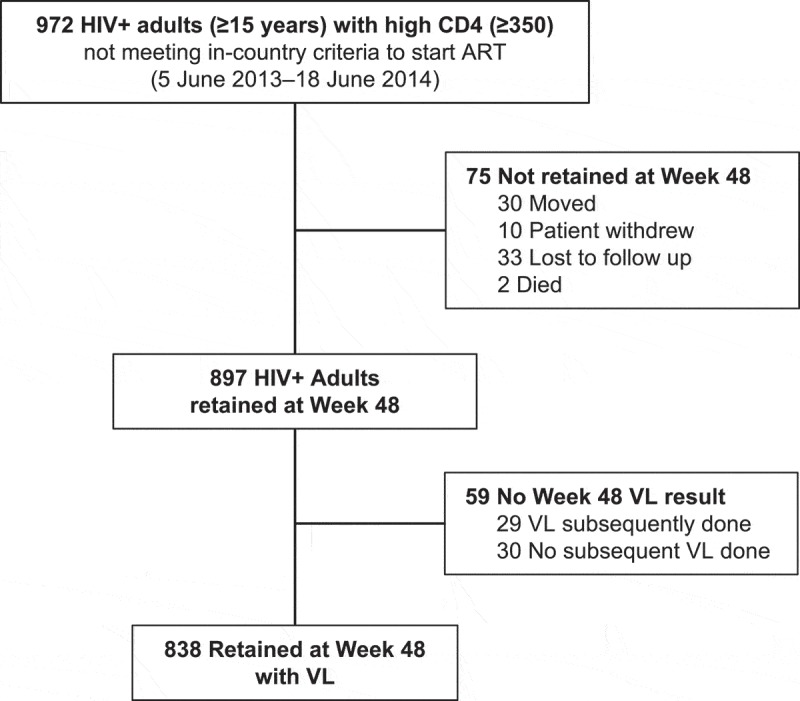
CONSORT diagram for adult participants. Analysis of 972 HIV-positive adults (age ≥ 15) with CD4+ count ≥350/μL who initiated ART.

**Figure 2.**
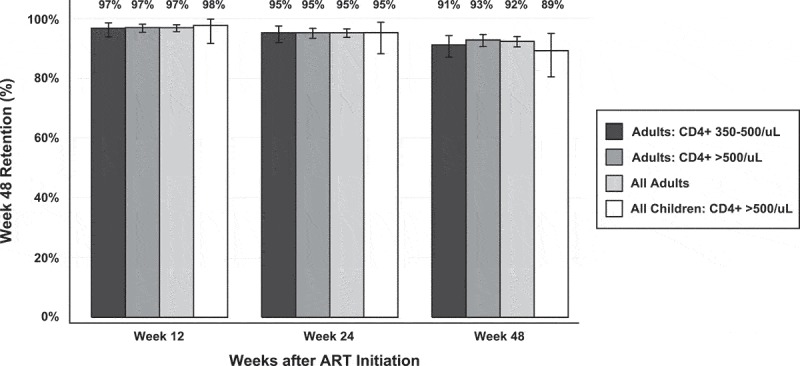
Retention in care at weeks 12, 24 and 48 following ART initiation among adults with CD4+ count 350–500/μL (dark grey), adults with CD4+ >500/μL (medium grey), all adults regardless of CD4+ count (light grey) and children (age < 15, CD4+ ≥500/μL; white), with 95% confidence intervals (black error bars).

Of the 972 adults who initiated ART, 897/972 were retained in care at week 48 (92%; Figure 1). The most common reasons for non-retention included moving away from study region (*n *= 30) and becoming lost to follow-up (*n *= 33). Retention patterns (i.e. retention rates at 12, 24 and 48 weeks) were similar in adults who initiated ART at CD4+ 350–500/μL versus >500/μL (Figure 2). Of 897 adults retained at week 48, 838/897 (93%) had a VL measurement available for analysis. Of these, VL was undetectable in 778/838 (93%; Table 3). We performed an intention-to-treat (ITT) analysis that considered adults not retained at week 48 (*n *= 75), as well as adults retained at week 48 but without VL measured (*n *= 30), to be virologically detectable. Adults retained at week 48 lacking VL s but who subsequently had a VL ascertained (*n *= 29) had virologic suppression assessed using the post-week 48 VL. Viral suppression in this ITT analysis showed 800/972 (82%) participants with viral suppression.

**Table 3.** HIV RNA suppression in adults (*n *= 838) and children (*n* = 71) 48 weeks after initiating antiretroviral therapy at high CD4+ counts

	Adults (≥fifteen years)						Children (two to fourteen years)	
	**CD4+ 350–500/μL** (*n* = 232)		**CD4+ >500/μL** (*n* = 606)		**All Adults** (*n* = 838)		**CD4+ >500/μL** (*n* = 71)	
HIV RNA (copies/mL)	*N*	%	*N*	%	*N*	%	*N*	%
<500	218	94.0	560	92.4	778	92.8	65	91.5
500–10,000	7	3.0	20	3.3	27	3.2	4	5.6
10,001–100,000	6	2.6	20	3.3	26	3.1	2	2.8
>100,000	1	0.4	6	1.0	7	0.8	0	0.0

### Adult adverse events

Overall, grade III/IV clinical and laboratory adverse events were rare. Among adults, 95 adverse events occurred in 74/972 (7.6%) of patients. The most common clinical adverse events were fever (*n *= 5 [malaria, *n *= 4, pneumonia, *n *= 1], all resolved) and dizziness (*n *= 2; related to EFV initiation, both resolved). The most common laboratory adverse event was neutropenia (*n *= 16, all asymptomatic and not associated with fever). Two patients died during the first 48 weeks on ART, one due to alcohol poisoning, and the other from causes we were not able to ascertain. Of the 74 patients who had either a clinical or laboratory event, the median CD4+ count at ART initiation was 711 cells/μL (IQR, 499–838 cells/μL). Alterations to ART medications were rare: overall, only 8/972 patients (0.8%) of patients switched from first-line EFV-based ART to second-line ritonavir/lopinavir-based ART. Reasons for ART switches included dizziness (*n *= 2), gynaecomastia (*n *= 2) and other (*n *= 4).

### Streamlined care parameters

A total of 26/510 (5.1%) of adult patients in Uganda study clinics received care that included both HIV services and services for non-communicable diseases (either hypertension or diabetes). Overall, clinicians in the 10 Uganda clinics received a total of 183 telephone calls from 510 patients in the first year after ART initiation (Supplementary Appendix B). In 84%, the caller was the patient; 9% of the time, the caller was a family member. Most calls occurred during normal business hours 9am–5pm (103/183, 56%), and only 7% occurred overnight (13/183). Most calls were 1–5 min in duration (127/183, 69%). Top reasons for calls included requests to clarify appointment dates (24% of calls), discussion of a perceived ART side effect (15%) and discussion of a non-urgent health problem (11%). Most frequent responses by clinicians included general counselling (24% of calls), clarification (22%), rescheduling (15%) or moving up (8%) of appointment dates, or other responses [mostly discussion or clarification of appointment dates or test results (Supplementary Appendix B)].

### Paediatric retention in care, virologic suppression and adverse events

**Figure 3.**
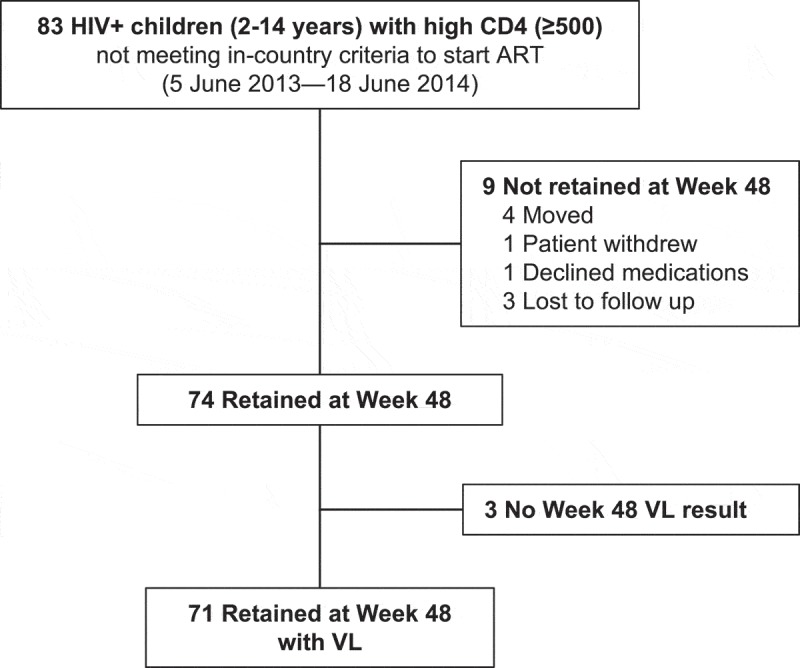
CONSORT diagram for paediatric participants. Analysis of 83 HIV-positive children (age < 15) with CD4+ count ≥500/μL who initiated ART.

As shown in Figure 3, 83 children (two to fourteen years) who did not meet in-country criteria to start ART and who had CD4 ≥ 500/μL initiated ART. ART regimens included (1) ABC + 3TC + EFV (*n *= 62/83 [74.7%]), (2) ABC + 3TC + NVP (*n *= 4/83 [4.8%]) and (3) TDF + (3TC or FTC) + EFV (*n *= 17 [20.5%]). Retention in care at week 48 was 89% (74/83; Figure 2): four children moved away, one withdrew consent, one declined medications and three had non-ascertained reasons for being lost to follow-up (Figure 3). Of 74 children retained at week 48, 71/74 (96%) had a measured VL, and virologic suppression was 92% (65/71; Table 3). Adverse events were uncommon: only 3/83 children (3.6%) had a clinical or laboratory adverse event of grade III/IV. Events were neutropenia (*n *= 1), thrombocytopenia (*n *= 1) and rash (*n *= 1). Two changes in ART occurred: nevirapine was stopped due to a grade IV hypersensitivity rash, and abacavir was stopped due to a grade II rash in ART regimen.

## Discussion

In a network of government-sponsored rural Ugandan and Kenyan healthcare clinics within the SEARCH Study, we assessed the effectiveness of a patient-centred streamlined care delivery model for adults and paediatric patients with high CD4+ counts. We found excellent rates of retention in care, virologic suppression and low rates of toxicity in both adult and paediatric patient groups. Our data represent one of the largest reports of a multicomponent streamlined care system designed for individuals with high CD4+ counts – a patient population whose numbers are growing in the current universal ART era, and who are likely to be the most amenable to streamlined efficient care systems.

The high rate of retention in care we observed may be partly attributable to our streamlined care system addressing three key domains that literature has shown to predict retention failures: (1) structural barriers, (2) suboptimal patient–clinician relationships and (3) gaps in patient and clinician knowledge that lead to stigma and motivation problems. First, structural barriers to care (e.g. long wait times, frequent visits, opportunity costs to work and inconvenience) have been repeatedly demonstrated to degrade retention in care [23–26]. Our streamlined care system aimed to reduce wait times using a nurse-driven rapid process focusing on basic ART toxicity screening [20,27]. We previously reported that visit lengths for streamlined care patients (including pre-visit wait time) were 68 min on average, compared to 155 min for non-streamlined care. This was due both to longer waits before and during visits [27]. Our streamlined care system also aimed to reduce visit frequency both by allowing longer three-month ART refills (vs. typical one to two month refills in Uganda and Kenya), as well as by combining healthcare provision for HIV, hypertension and diabetes into single visits when applicable.

Second, patient perceptions of mistreatment by clinicians have also been clearly linked to retention in care failures [25,26]. Patients cite judgmental, negative interactions with clinicians during discussions of adherence, ART use, missed visits or gaps in care. Our streamlined care system thus aimed to formally train clinicians in providing friendly, welcoming care using supportive, encouraging interactions revolving around patients’ desires for their care. This was especially important when clinicians faced challenging situations of incomplete ART adherence or missed visits. We also incorporated regular team discussions for clinicians to share challenges and successes – a feedback process that may have helped sustain a positive environment. Literature on promoting patient-centred care supports the concept that such practice can be taught [28], promotes clinician job satisfaction and reduces burnout that lowers quality of care over time [29].

Third, improvements in retention in care have been linked to increased knowledge and understanding of HIV disease and ART [30]. More broadly, the importance of patient knowledge and health literacy has gained attention as a crucial factor for durable retention in care – an outcome that depends on patients building self-efficacy, and feeling knowledgeable enough to participate in shared decision-making [31]. In addition, stigma is well established in the literature as a persistent threat to retention in care [25]. Our streamlined care system attempted to reduce stigma by embedding HIV care within a broader streamlined general medical care system, offering hypertension, diabetes and other care in parallel with HIV care. This may have allowed patients to visit the clinic without the reason being apparent to others (i.e. because all services [both HIV-related and non-HIV-related] were delivered jointly). Further, by labelling our clinics as “medical” rather than “HIV” clinics, stigma may have been further reduced. Lastly, since HIV-positive patients often have longer visits than patients receiving non-HIV-related care, our short visit times – on average 30 min [20,27] – may have eliminated this differentiating factor that could identify patients’ HIV diagnoses. In our view, the streamlined care components that impacted outcomes most strongly were the rapid visit procedures that resulted in <30 min visits, combined with long three-month ART refills (both of which lowered opportunity costs for attending clinic visits), as well as the patient-centred environment and direct discussions of VL results. Qualitative analyses of our patients’ perceptions on structural barriers, patient–clinician relationships and motivation and stigma are already underway and will help determine which features of streamlined care were most effective.

We observed high rates of viral suppression, consistent with a growing literature showing positive outcomes in ART patients, including those with high CD4+ T cell counts >350–500/μL [2–4]. Recent population-based cohorts have shown high viral suppression among adults with lower CD4+ counts receiving ART under standard, non-streamlined/differentiated care models. Viral suppression has been reported from cohorts in Nigeria (85% with VL ≤ 400 c/mL) [32], Malawi (91% with VL ≤ 1000 c/mL) [33] and Kenya (84% with VL ≤ 1000 c/mL) [34]. In addition, our group reported an 82% viral suppression (VL ≤ 500 c/mL) in the large rural Ugandan and Kenyan populations comprising the SEARCH Study baseline population [35]. Thus, although we cannot attribute a causal link between our streamlined care intervention and our one-year results in this report, our viral suppression rates among patients with high CD4+ counts are similar to or exceed those seen in lower CD4+ count patients, most of whom did not receive streamlined/differentiated care.

Our structured VL counselling may have influenced our positive results: we shared VL values with patients using a system that emphasized knowledge and linkages between ART adherence and VL results. We observed that sharing undetectable VL results allowed reinforcement of patients’ good adherence patterns. We also found that numerically demonstrating detectable viremia was a powerful tool for building patients’ knowledge, promoting better adherence and sustaining adherence over time. As VL access continues to expand, coupling testing with structured counselling will be key to maximizing its impact.

The HIV-infected children in this study achieved excellent 48-week retention (89%) and virologic suppression (92%) rates compared to other reports from African settings. In one study of 4803 South African children, 66% of children receiving a community-based intervention for ART support were virally suppressed compared to 55% receiving standard of care [36]. In cross-sectional data from programmes in Swaziland, 71% of children <ten years old were virologically suppressed [37]. HIV-infected children and their guardians receiving care in rural clinics face many of the same barriers as adults that were targeted by our streamlined care model (i.e. structural barriers, challenging clinician–patient relationships and knowledge gaps enabling stigma and motivation problems). However, HIV-infected children face additional systems-level challenges, including the complexity of weight-based ART dosing and shifting age- and CD4-specific ART-initiation thresholds [38]. For this reason, centres with paediatric HIV specialization are often developed in high prevalence urban areas. However, our results suggest that as universal ART programmes continue to decentralize and expand to rural populations, excellent outcomes can be achieved by integrating paediatric HIV care with adult programmes using streamlined care models like that employed by SEARCH.

Key considerations during adoption of streamlined care models include their costs, and the potential opportunities and challenges in their scale up throughout sub-Saharan Africa [39]. We recently estimated costs of our streamlined care model to be $275 per person per year of ART delivery [27], placing it in line with or less expensive than other care models, some of which do not include VL testing and counselling. These costs may decrease over time as ART costs are further optimized, and as VL testing grows and achieves greater economy of scale. Challenges to scaling up streamlined care may include barriers in rapidly shifting the public health-centred model of clinic care to a truly patient-centred paradigm, the need to secure excellent training and mentoring for clinicians at all levels, instituting methods of real-time tracking of the quality of care and providing meaningful feedback within health systems and securing durable funding for the continued expansion of VL testing. However, opportunities to use the platform of HIV care delivery to improve service delivery for a variety of other medical conditions are now being captured at an increasing rate. Streamlined care models designed to prioritize patients’ experience and deliver broad health outcomes have great potential to accelerate this progress.

Our study was subject to several limitations. First, we were not able to ascertain VL results on all participants; however, as our intention-to-treat analysis showed, viral suppression remained high after conservatively accounting for these missing values. Second, because we delivered patient-centred streamlined care as a multicomponent package intervention, we could not assess which specific components of the streamlined care system are the most central to its overall success. However, since most of our streamlined care components are low cost (or even cost negative, as with providing longer ART refills that lengthen visit intervals and reduce daily patient loads), deploying streamlined care as a package may be compelling at a public health level. Such packages could be customized to maximize effectiveness in different care settings where varying components play different roles in achieving retention in care and viral suppression. Qualitative analyses are underway to assess patient and clinician perspectives on elements of streamlined care and reasons for success. Third, the use of mobile phone communication, both outgoing (for appointment reminder calls) and incoming (providing telephone access to patients), relies on patients having reliable access to a mobile phone. This may limit generalizability of our results to unselected patient groups. However, mobile phone use is expanding rapidly in both Uganda and Kenya [40,41], and mobile phone-based interventions are gaining traction as inexpensive and easy ways to deliver interventions [15,42], including to children and adolescents [43]. Finally, the limited numbers of paediatric patients in our analysis limit the precision of our estimates of viral suppression, and their generalizability to other sub-Saharan African settings.

## Conclusions

In a network of prototypic healthcare clinics in rural Ugandan and Kenyan communities, we demonstrate excellent retention in care, viral suppression and low rates of adverse events among HIV-positive adults and children with high CD4+ counts receiving ART via a streamlined care delivery system. Our results amplify growing evidence that streamlined care models, integrating paediatric and adult care in rural clinics, can boost the efficiency of the healthcare delivery system while also becoming more patient centred. Such efforts will be central to meeting global goals of universal ART coverage for all HIV-positive individuals in the coming years.

## Notes

To access the supplementary material to this article please see Supplementary Files under Article Tools online.
